# Transcriptome Comparison Reveals Distinct Selection Patterns in Domesticated and Wild Agave Species, the Important CAM Plants

**DOI:** 10.1155/2018/5716518

**Published:** 2018-11-22

**Authors:** Xing Huang, Bo Wang, Jingen Xi, Yajie Zhang, Chunping He, Jinlong Zheng, Jianming Gao, Helong Chen, Shiqing Zhang, Weihuai Wu, Yanqiong Liang, Kexian Yi

**Affiliations:** ^1^Environment and Plant Protection Institute, Chinese Academy of Tropical Agricultural Sciences, Haikou 571101, China; ^2^National Key Laboratory of Crop Genetic Improvement, Huazhong Agricultural University, Wuhan, Hubei 430070, China; ^3^Hainan Climate Center, Haikou 570203, China; ^4^Institute of Tropical Bioscience and Biotechnology, Chinese Academy of Tropical Agricultural Sciences, Haikou 571101, China

## Abstract

Agave species are an important family of crassulacean acid metabolism (CAM) plants with remarkable tolerance to heat and drought stresses (*Agave deserti*) in arid regions and multiple agricultural applications, such as spirit (*Agave tequilana*) and fiber (*Agave sisalana*) production. The agave genomes are commonly too large to sequence, which has significantly restricted our understanding to the molecular basis of stress tolerance and economic traits in agaves. In this study, we collected three transcriptome databases for comparison to reveal the phylogenic relationships and evolution patterns of the three agave species. The results indicated the close but distinctly domesticated relations between *A. tequilana* and *A. sisalana*. Natural abiotic and biotic selections are very important factors that have contributed to distinct economic traits in agave domestication together with artificial selection. Besides, a series of candidate unigenes regulating fructan, fiber, and stress response-related traits were identified in *A. tequilana*, *A. sisalana*, and *A. deserti*, respectively. This study represents the first transcriptome comparison within domesticated and wild agaves, which would serve as a guidance for further studies on agave evolution, environmental adaptation, and improvement of economically important traits.

## 1. Introduction

Agave species assembled an important group of crassulacean acid metabolism (CAM) plants with remarkable tolerance to heat and drought stresses in arid regions [1]. CAM plants usually have a higher efficiency in water use than C3 and C4 plants [2]. For this reason, CAM plants brought a great chance to enhance sustainable production of food and bioenergy under the background of limited freshwater resources and global climate change [3]. As a traditional cultivated CAM plant, *Agave tequilana* has been used for the production of distilled spirit tequila for centuries [4]. Further, it also shows a great potential in bioenergy production [5]. Besides, *Agave sisalana* has also been widely cultivated as a cash crop for fiber production in tropical regions [6]. As a native wild plant in the Sonoran Desert regions of the Southwestern United States and Northwestern Mexico, *Agave deserti* has successfully survived in a severe environment within elevation ranges that experience both hot, dry summers and occasional freezing temperatures in winter [7, 8]. This capacity of high tolerance to multiple stresses has important values to the improvement of the main food crops. These economic and stress-tolerant features have made agave a model CAM crop system for a hot and dry/droughty/xeric environment [9].

However, few reports have revealed the physiology and molecular basis of agaves, especially in *A. sisalana*. To date, *A. tequilana*-related studies mainly focused on fructan, with the obvious purpose of improving fructan production and application to generate bioenergy [5, 10, 11]. *A. deserti*-related studies were mainly conducted for its ecological and physiological adaptation to a severe environment, which is highly valuable for the improvement of the main food crops [12]. Besides, a series of saponin-related researches have been reported, while few reports were related to fiber in *A. sisalana* [13, 14]. These three agave species are closely related species but with different remarkable biological features. A previous study has revealed the phylogenetic relations according to their trnL sequences [15]. The result showed that they were closely related species in spite of different origins for *A. tequilana* (Jalisco), *A. deserti* (the Sonoran Desert), and *A. sisalana* (Chiapas) [4, 6, 7]. Agaves have very large genomes, which has significantly restricted their genome assembly and limited our understanding to their evolution pattern [16]. In other crops, accessible genomes and genome-wide association analysis have revealed many economically important traits for crop improvement [17–19]. Recently, the development of next-generation sequencing has brought a new direction for gene-related studies without the restriction of genome data [20, 21]. Furthermore, transcriptome comparison has also been conducted for evolution analysis and searching economically important traits in some genome unavailable crops [22]. In this study, we selected three transcriptome databases for comparison to reveal the phylogeny and evolution pattern of the three agave species [23, 24]. Those genes related to species-specific traits would be also identified and evaluated for their importance in agronomy production and environmental adaptation of agaves. This study represents the first transcriptome comparison within domesticated and wild agaves, which would serve as a guidance for further studies on agave evolution, environmental adaptation, and improvement of economically important traits.

## 2. Materials and Methods

### 2.1. Phylogenetic Analysis

Phylogenetic analysis was conducted by MEGA 5.0 software with the minimum-evolution method [25]. The methods and parameters were according to the previous study [15]. The bootstrap method was employed for confidence in nodes with 1000 replicates. Partial chloroplast sequences for *A. tequilana* and *A. deserti* were downloaded from NCBI (https://www.ncbi.nlm.nih.gov/) with the accession numbers GAHU01110124 and GAHT01022741, respectively. Partial chloroplast sequences for *A. sisalana* were obtained from a previous transcriptome database [24]. The whole chloroplast sequence of *A. americana* was obtained from NCBI under the accession number KX519714. The trnL+trnL-trnF sequences (about 900 bp) for the 4 and other 14 agave species were also downloaded according to the previous study [15].

### 2.2. Transcriptome Data and Gene Annotation

Three agave transcriptome databases were according to previous studies [23, 24]. Transcriptome assembly of the Illumina sequence was performed by Rnnotator for each species, respectively [26]. Only unigenes with corresponding predicted proteins in *A. deserti* transcriptome were used to search orthologous genes from *A. tequilana* and *A. sisalana* transcriptomes by the BBH method, respectively [27]. These orthologous unigenes in three transcriptomes were annotated in the public databases: NCBI nonredundant protein (Nr) and nonredundant nucleotide (Nt) databases (http://www.ncbi.nlm.nih.gov/), Swiss-Prot (http://www.ebi.ac.uk/uniprot/), and Gene Ontology (GO) (http://www.geneontology.org/), respectively.

### 2.3. Ka/Ks Analysis

It represents positive selection when the ratio of nonsynonymous (Ka) to synonymous nucleotide substitutions (Ks) is significantly higher than 1, whereas the ratios significantly less than or equal to 1 are subjected to purifying or neutral selection [28]. The Ka, Ks, and Ka/Ks values were estimated by the Codeml model of the program of phylogenetic analysis by maximum likelihood (PAML) between *A. deserti* unigenes with orthologous unigenes in *A. tequilana* or *A. sisalana*, respectively [29].

### 2.4. In Silico Gene Expression Analysis

The expression pattern of positive selected unigenes was subjected to in silico gene expression analysis in agave leaves. The reads per kilobase per million mapped read (RPKM) value of these unigenes in *A. deserti* and *A. tequilana* were calculated by RSEM software according to the previous study [23, 30]. The RPKM data was further normalized with two reference genes (tubulin and serine/threonine-protein phosphatase) in each agave species [31].

### 2.5. Selection Pressure Detection and Protein Structure Modeling

To detect the selection pressure on positive selection unigenes, Ka/Ks ratios were calculated in sliding window (30 bp under a step size of 6 bp) by using DnaSP 5.0 [32]. Translated protein sequences of positive selection unigenes were used for structure modeling by Swiss-Model (https://swissmodel.expasy.org/) [33].

## 3. Results

### 3.1. Phylogeny of Agave Species

**Figure 1:**
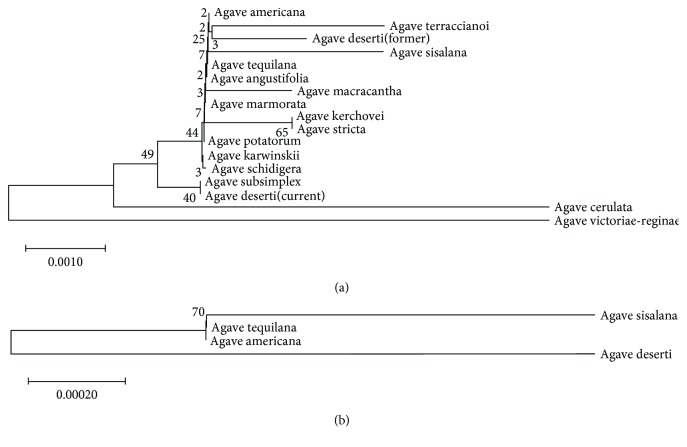
Phylogenetic analysis of chloroplast sequences in Agave species. (a) The trnL+trnL-trnF sequences (about 900 bp) were used to construct a phylogenetic tree. (b) The sequences of *A. tequilana* (GAHU01110124), *A. deserti* (GAHT01022741), and *A. americana* (KX519714) were from NCBI. *A. sisalana* (CL7065.Contig2) sequence and the other 13 sequences were from previous studies [15, 24].

Phylogenetic analysis was conducted to reveal the phylogenic relation for agave species in this study. We obtained chloroplast sequences for *A. tequilana* (GAHU01110124), *A. sisalana* (CL7065.Contig2), and *A. deserti* (GAHT01022741), by using blast against three agave transcriptomes [23, 24]. The chloroplast sequence of *A. americana* was from GenBank under the accession number KX519714 (http://www.ncbi.nlm.nih.gov/genbank/). The trnL+trnL-trnF sequences (about 900 bp) from the 4 and other 13 agave species [15] were employed for phylogenetic analysis to reveal their evolutionary pattern. The results indicated that *A. tequilana*, *A. sisalana*, *A. americana*, and *A. deserti* (former, DQ500894 + DQ500928) sequences were grouped together, while the *A. deserti* (GAHT01022741) sequence was in another group (Figure 1(a)). We further selected the four chloroplast sequences (about 2490 bp) for phylogenetic analysis and found that *A. deserti* (GAHT01022741) was separated with the other 3 species (Figure 1(b)).

### 3.2. Sequence Comparison between Agave Transcriptomes

**Figure 2:**
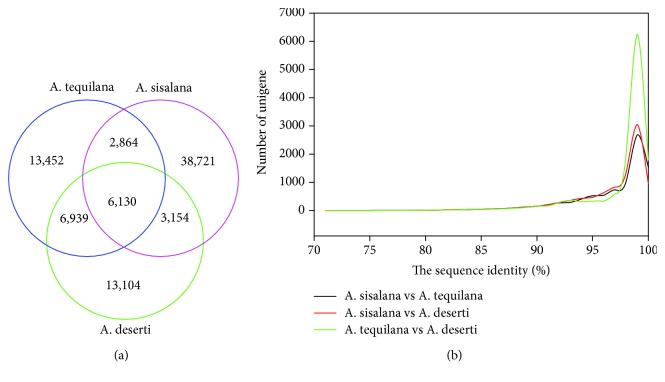
Sequence comparisons between domesticated *A. tequilana* and *A. sisalana* and wild *A. deserti* transcriptomes. (a) The sketch map showing 6130 unigene terms that were identified within the three transcriptomes. (b) The identity distribution of all orthologous unigene pairs.

**Figure 3:**
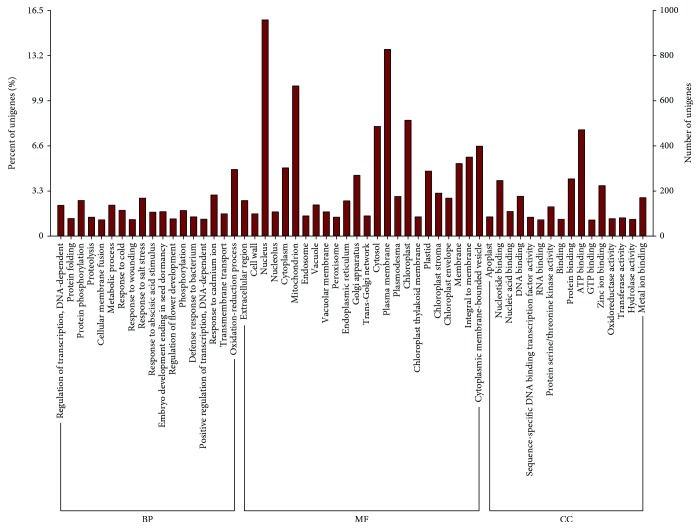
Gene ontology classifications of 6130 orthologous unigenes. The results are clustered in the three main categories as biological process (BP), cellular component (CC), and molecular function (MF).

A summary for the three databases was described in Table 1. There were 29,367, 29,327, and 50,851 sequences employed for orthologous gene searching from *A. tequilana*, *A. deserti*, and *A. sisalana* transcriptomes, respectively. As a result, we identified 13,069 unigene pairs between *A. tequilana* and *A. deserti*, 8976 pairs between *A. sisalana* and *A. tequilana*, and 9284 pairs between *A. sisalana* and *A. deserti* (Figure 2(a)). Among these orthologous unigene pairs, more than 91% unigene pairs had an identity over 91% (Figure 2(b)). Furthermore, a total of 6130 unigene terms were obtained from the three agave transcriptomes. GO functional classification indicated that these genes were assigned to 30,405 functional terms. There were 14,915 terms in biological process (49.05%), 8546 in molecular function (28.11%), and 6944 in cellular component (22.84%) (Figure 3).

### 3.3. Identification of Genes Selected in the Domestication of Agaves

**Figure 4:**
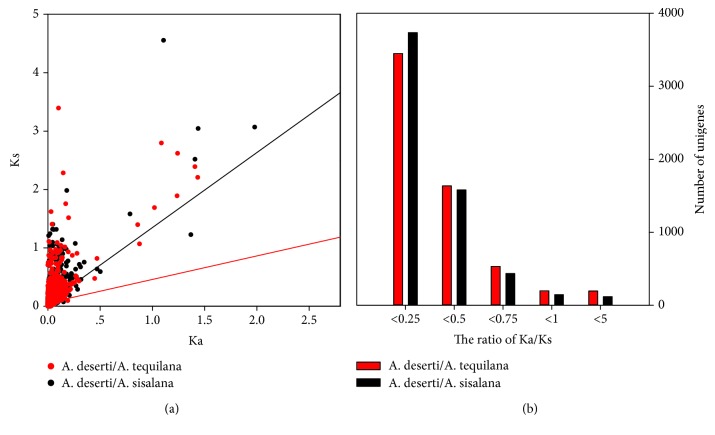
The nonsynonymous (Ka) and synonymous (Ks) nucleotide substitutions, as well as their Ka/Ks ratio. (a) The scatter diagram of Ka and Ks values of *A. tequilana* (red) and *A. sisalana* (black) compared with *A. deserti*, respectively. (b) The Ka/Ks distribution of *A. tequilana* (red) and *A. sisalana* (black) compared with *A. deserti*, respectively. The *x*-axis indicates the ratio of Ka/Ks.

The Ka, Ks, and Ka/Ks values were calculated for 6130 orthologous unigene pairs in *A. tequilana* and *A. sisalana* separately with *A. deserti* (Figure 4(a)). The correlation between Ka and Ks values was also estimated in *A. tequilana* (*r* = 0.515, *P* < 0.05) and *A. sisalana* (*r* = 0.206, *P* < 0.05) unigene pairs, respectively. 393 unigenes (6.5%) in *A. tequilana* and 262 unigenes (4.5%) in *A. sisalana* showed a Ka/Ks ratio higher than 1, while the Ka/Ks ratio of more than 90% unigenes was lower than 1 (Figure 4(b)).

The significance of the Ka/Ks value for all 6130 unigene terms was analyzed, and the results indicated that 1117 unigenes were significantly selected at least in *A. tequilana* or *A. sisalana* (*P* value < 0.05) (Supplementary Table 1). These genes were further characterized into 15 classifications according to their annotations (Table 2). Among these, 54 unigenes were subjected to positive selection (Ka/Ks > 1), while the residues of 1064 unigenes were subjected to purifying selection (Ka/Ks < 1). Furthermore, 27 and 19 unigenes were positively selected in *A. tequilana* and *A. sisalana*, respectively. 8 unigenes were positively selected in both agave species (Supplementary Table 1).

We further characterized the 54 unigenes subjected to positive selection and found that three unigenes were annotated as disease resistance protein (Table 3). In *A. tequilana*, four unigenes were annotated as 6G-fructosyltransferase, d-2-hydroxyglutarate dehydrogenase, gluconokinase, and 6-phosphofructokinase, respectively, and they might be related to fructan production, while in *A. sisalana*, five unigenes were characterized as auxin-responsive protein IAA6, gibberellin receptor, PHD and RING finger protein, elongation of fatty acids protein, and zinc finger A20 and AN1 protein, respectively. These unigenes are probably related to the fiber development in *A. sisalana*. Besides, two unigenes, designated as RING-H2 finger protein and TOM1-like protein, were positively selected in both *A. tequilana* and *A. sisalana*.

### 3.4. In Silico Expression of Genes under Positive Selection in Agave Species

**Figure 5:**
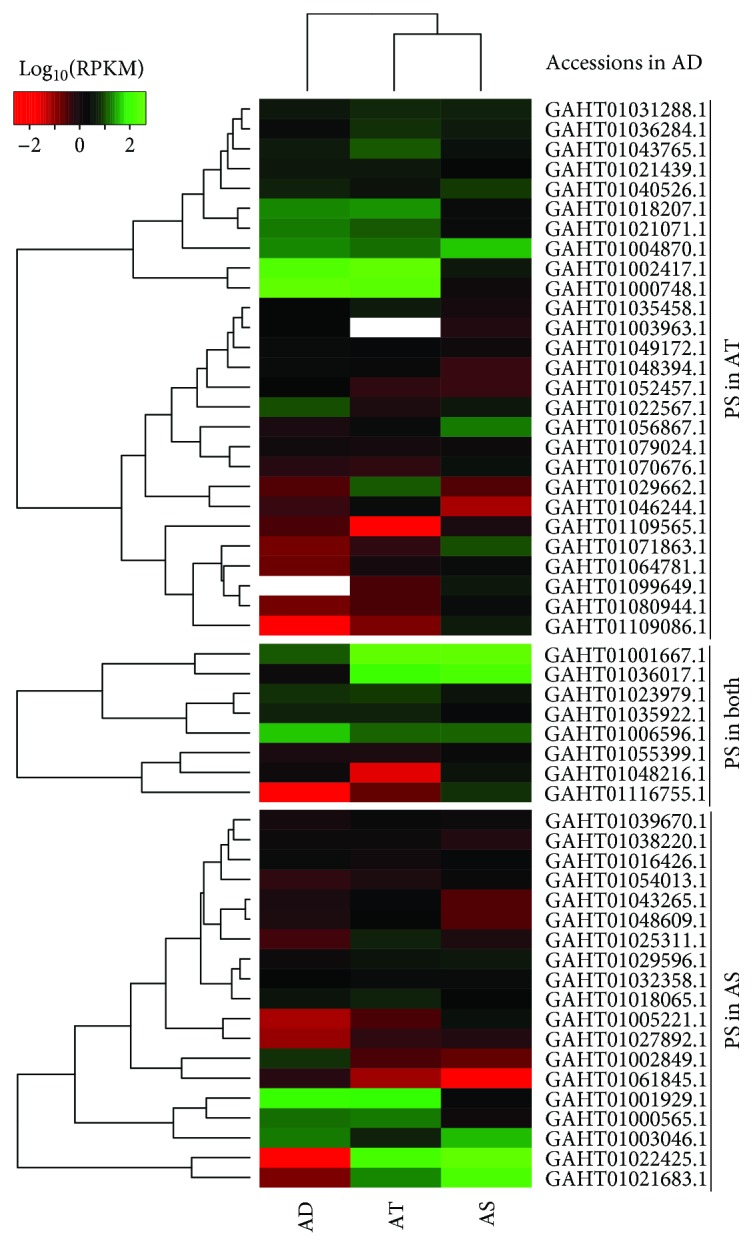
In silico expression pattern of positive selection (PS) unigenes in Agave leaves. Rows and columns in heat maps represent genes and Agave species. Species name of *A. deserti* (AD), *A. tequilana* (AT), and *A. sisalana* (AS) is shown under the heat maps. The colour bar at the top indicates the degree of expression: red—low expression; green—high expression.

The expression patterns of genes under positive selection were analyzed according to the three transcriptome databases. In agave leaves, the 27 and 8 unigenes were grouped into two expression modes (Figure 5). However, the 19 unigenes in *A. sisalana* were not distinctly clustered into different expression modes. All these genes were differentially expressed in *A. tequilana* or *A. sisalana* when compared with *A. deserti*.

### 3.5. Selection Pressure and Structure Model of Putative Economic Trait-Related Genes

**Figure 6:**
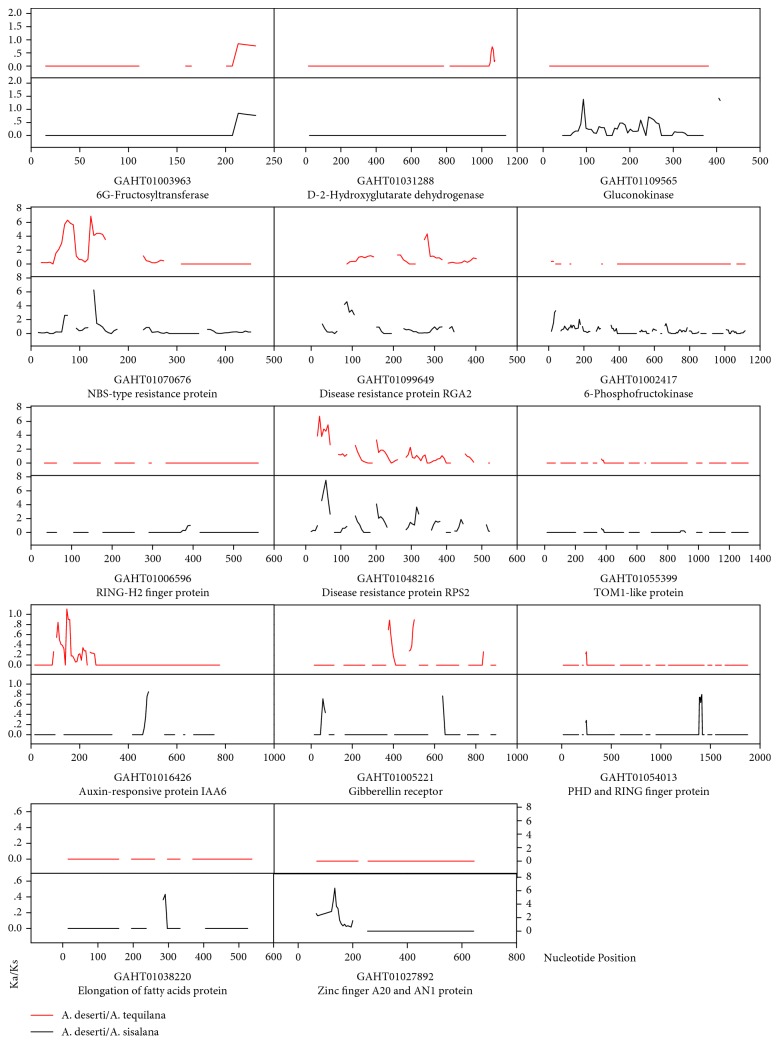
Sliding window analysis of positive selection unigenes in *A. tequilana* (red) and *A. sisalana* (black) compared with *A. deserti*, respectively. The window size is 30 bp, with a step size of 6 bp. The *x*-axis denotes the nucleotide position. The *y*-axis denotes the Ka/Ks ratio. The gaps represent Ka/Ks ratios that could not be computed.

The sliding window analysis was used to examine the selection pressure of putative economic Trait-related genes. The results indicated the existence of different selection pressures in *A. tequilana* and *A. sisalana* genes (Figure 6). The three disease resistance genes all had a strong selection pressure in most sequence regions. Five unigenes showed a stronger selection pressure in *A. sisalana* (GAHT01109565, GAHT01002417, GAHT01054013, GAHT01038220, and GAHT01027892). Only GAHT01031288 showed a stronger selection pressure in *A. tequilana*. The residue of five unigenes shared a strength-similar but region-different selection pressure.

**Figure 7:**
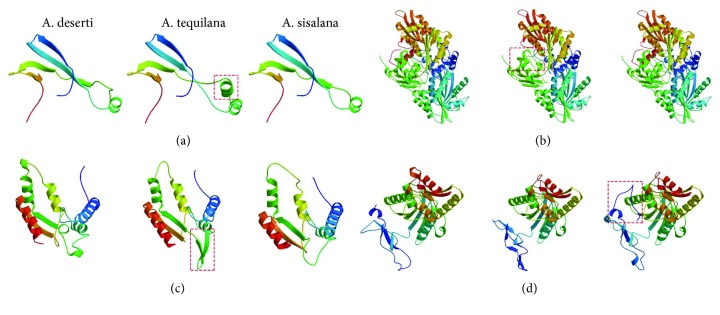
Protein structure model of 6G-fructosyltransferase (a), d-2-hydroxyglutarate dehydrogenase (b), gluconokinase (c), and 6-phosphofructokinase (d) in three Agave species by using Swiss-Model [33]. The significant structure difference within species was marked in red dotted line.

The structure modeling for the 14 unigenes was further conducted to analyze the difference of agave proteins. According to the results, four unigenes designated as 6G-fructosyltransferase (Figure 7(a)), d-2-hydroxyglutarate dehydrogenase (Figure 7(b)), gluconokinase (Figure 7(c)), and 6-phosphofructokinase (Figure 7(d)), could match Swiss-Model sequences with identity > 30%, coverage > 75%, and appropriate description (Supplementary Table 2). Their structure models also showed significant differences in *A. tequilana* or *A. sisalana*. The unigene with a significantly distinct structure was differentially expressed when compared to their orthologous unigenes in other two species (Figure 5).

## 4. Discussion

A previous phylogenetic analysis suggested that *A. deserti* was closely related to *A. angustifolia* [15]. However, we compared the recently published chloroplast sequence (GAHT01022741) with the former one (DQ500894 + DQ500928) in *A. deserti* and found them having totally different sequences. It might be caused by sample collection because the two studies were separately conducted in Mexico and America. Wild agave species are usually identified by morphological traits, which is not as reliable as molecular identification. Our phylogenetic results also indicated a different evolution relation by both short and long chloroplast sequences (Figure 1). This indicated the closer evolution relationship between *A. tequilana* and *A. sisalana* than the evolution relationship between *A. tequilana* and *A. deserti* or between *A. sisalana* and *A. deserti*. It has been reported that *A. tequilana* and *A. sisalana* both had genetic relations with *A. angustifolia* [6, 7]. The phylogenetic result has also proved that, however, the two species actually possessed very different agronomic traits and applications. An important reason should be artificial selection even if the low-frequency serendipitous backyard hybridization would lead to distinct domestication of crops. This is a historical inheritance in Mexico and has significantly enriched the genetic diversity of crops [34].

Agave species originate from Central America with high tolerance to drought and temperature, which makes them a main and important kind of plants there [9]. Therefore, they would inevitably confront a series of abiotic and biotic stresses. Actually, we identified 62, 127, and 77 unigenes with classifications of disease/defense, signal transduction, and transcription factor, respectively. Among them, 13 significant selected unigenes were related to disease resistance and ten of them subjected to purifying selection (Supplementary Table 1). This might be accompanied with the process of agronomic Trait-derived domestication. Many disease resistance genes were also found to be lost during domestication in other crops [17, 22]. A previous study has already revealed a differentiated selection pressure on NBS-LRR genes in some agave species [35]. In this study, the sliding window analysis also showed a strong selection pressure with the three disease resistance unigenes (Figure 6). Furthermore, two of the three unigenes (GAHT01070676 and GAHT01099649) were subjected to purifying selection in *A. sisalana* (Table 3), which might be harmful for growth and development. This might also be responsible for the susceptibility to zebra disease caused by *Phytophthora nicotianae* in *A. sisalana* [36].

It has been reported that several transcription factor families play an important role in abiotic stress regulation, such as bHLH, zinc finger, MYB, AP2, NAC, WRKY, and bZIP families [37–42]. We also found 47 TFs subjected to purifying selection either in *A. tequilana* or in *A. sisalana*, from the bHLH (8), zinc finger (23), MYB (6), AP2 (5), NAC (3), WRKY (1), and bZIP (1) families (Supplementary Table 1). For agave species, drought and high temperature are the main abiotic stresses. We speculated that different habitats should be an important natural selection pressure that affected the shape and size of the three agave species [43]. The purifying selection of the 47 stress-related candidate TFs might weaken the drought tolerance of *A. tequilana* and *A. sisalana* but enhance their biomass accumulations. The complex regulation and interaction of these TFs might be the key to reveal the mechanism of the remarkable drought tolerance in *A. deserti*. Much more molecular characterizations are still needed in future studies.

It has been reported that fruit/seed-related traits were subjected to high artificial selection pressure for their economic value [18, 19]. In *A. tequilana*, the most important economic trait focuses on fructan, and several studies have conducted functional characterization for fructosyltransferase genes [10, 11, 44]. In this study, we identified a fructan-related unigene and it was subjected to positive selection in *A. tequilana* (Table 3). Besides, three carbohydrate-related unigenes were also subjected to positive selection. Their positive selection might be responsible for the improvement of fructan yield in *A. tequilana*. In *A. sisalana*, fiber is the main economic purpose for its cultivation in tropical areas. In the present study, we found 5 unigenes subjected to positive selection only in *A. sisalana* (Table 3). A previous publication has reviewed the hormonal regulation of secondary cell wall formation [45]. The zinc finger family TFs are also proved to regulate cell wall development and cellulose biosynthesis [46, 47]. Besides, the elongation of fatty acids protein plays an important role in cell elongation [48]. Therefore, we speculated that the fiber-related traits in agaves are more likely controlled by hormonal and transcriptional regulation. And there are significant differences when compared with fructan-related traits, which might be mainly controlled by metabolic regulation in *A. tequilana*. Natural fiber is commonly generated as the result of secondary cell wall thickening in the main fiber crops such as cotton, ramie, flax, and hemp [49–52]. As a constitutive structure of plant cells, there are many housekeeping and regulating genes during cell wall development, especially the secondary cell wall development [45, 53–55]. In contrast, fructans are mainly responsible for carbohydrate storage to vegetative tissues in many plant species [56]. They have also been increasingly considered protective agents against abiotic stresses [57]. However, the capacity to produce and store fructans in *A. tequilana* is much stronger than that in *A. deserti*, even if there is a much more severe environment for *A. deserti* [23, 44]. It is probably because fructan-related traits are regulated at the metabolic level. A more recent study has combined transcript, protein, and metabolite methods to reveal the molecular basis of the CAM process in agave [58]. The rapid development of high-throughput molecular methods has brought a great opportunity for the further understanding of the differences and evolution patterns among agave species.

## 5. Conclusion

This study represents the first transcriptome comparison within domesticated and wild agave species. The results revealed the importance of abiotic/biotic natural selection in agave evolution. Four unigenes related to fructan in *A. tequilana* and five unigenes related to fiber in *A. sisalana* were positively selected. These genes revealed the difference between *A. tequilana* and *A. sisalana* evolution, which would serve as a guidance for further studies on agave evolution, environmental adaptation, and improvement of economically important traits.

## Figures and Tables

**Table 1:** Summary of the transcriptomes for the 3 Agave species.

Species	*A. tequilana*	*A. deserti*	*A. sisalana*
Total sequenced high-quality data	293.5 Gbp	184.7 Gbp	11.3 Gbp
Number of Agave unigenes	204,530	128,869	131,422
Sum length of Agave unigenes	204.9 Mbp	125.0 Mbp	77.6 Mbp
N50 length	1387 bp	1323 bp	861 bp

**Table 2:** Characterization of significant selection unigenes.

Classification	Significant selection unigenes			
	Total	In *A. sisalana*	In *A. tequilana*	In both
Cell growth/division	78	30	37	11
Cell structure	37	12	22	3
Disease/defense	62	25	22	15
Energy	26	5	18	3
Intracellular traffic	6	2	3	1
Primary metabolism	94	27	55	12
Protein destination and storage	102	27	56	19
Protein synthesis	28	7	16	5
Secondary metabolism	56	16	33	7
Signal transduction	127	40	64	23
Transcription	82	26	40	16
Transcription factor	77	28	37	12
Transporter	56	17	31	8
Transposon	9	3	4	2
Unclassified	277	72	161	44
Total	1117	337	599	181

**Table 3:** Unigenes subjected to positive selection in the domestication of Agave species.

*A. deserti* accession	*A. tequilana*				*A. sisalana*				Functional annotation
	Accession	Ka	Ks	Ka/Ks	Accession	Ka	Ks	Ka/Ks	
GAHT01003963	GAHU01106881	0.0533	0.0493	1.0803	Unigene21947	0.0084	0.0104	0.8112	6G-Fructosyltransferase
GAHT01031288	GAHU01044798	0.0133	0.0121	1.1055	Unigene12882	0.0011	0.0269	0.0428	d-2-Hydroxyglutarate dehydrogenase
GAHT01109565	GAHU01096053	0.0165	0.0084	1.9594	CL19921.Contig1	0.0444	0.2201	0.2015	Gluconokinase
GAHT01002417	GAHU01001324	0.0214	0.0106	2.0164	Unigene18337	0.0639	0.1531	0.4171	6-Phosphofructokinase
GAHT01070676	GAHU01114859	0.0876	0.0797	1.0994	Unigene374	0.0929	0.2571	0.3613	NBS-type resistance protein RGC5
GAHT01099649	GAHU01173424	0.1314	0.1049	1.2523	CL12994.Contig3	0.1753	0.2373	0.7387	Disease resistance protein RGA1
GAHT01006596	GAHU01010948	0.0167	0.0132	1.2634	CL13186.Contig4	0.023	0.0139	1.6485	RING-H2 finger protein
GAHT01048216	GAHU01097154	0.1621	0.1363	1.1894	CL18667.Contig2	0.149	0.0737	2.0217	Disease resistance protein RPS2
GAHT01055399	GAHU01077165	0.0118	0.0043	2.7653	Unigene28650	0.0188	0.0065	2.9139	TOM1-like protein
GAHT01016426	GAHU01038186	0.0143	0.1124	0.1272	CL8882.Contig1	0.0289	0.0226	1.28	Auxin-responsive protein IAA6
GAHT01005221	GAHU01003524	0.0322	0.0583	0.5528	CL2916.Contig1	0.0301	0.0107	2.8275	Gibberellin receptor
GAHT01054013	GAHU01067136	0.009	0.0092	0.9716	Unigene233	0.0081	0.0059	1.383	PHD and RING finger protein
GAHT01038220	GAHU01063926	0.0116	0	—	Unigene4300	0.0156	0.0049	3.154	Elongation of fatty acids protein
GAHT01027892	GAHU01009883	0.0022	0.0281	0.0794	Unigene28305	0.0659	0.0551	1.1956	Zinc finger A20 and AN1 protein

## Data Availability

The data used to support the findings of this study are available from the corresponding author upon request.

## Supplementary Materials

Supplementary 1 Table S1: the results of classification, Ka/Ks analysis, and functional annotation for the 6130 unigenes.

Supplementary 2 Table S2: details for structure modeling of putative economic Trait-related genes.

## References

[1] Borland A. M., Griffiths H., Hartwell J., Smith J. A. C. (2009). Exploiting the potential of plants with crassulacean acid metabolism for bioenergy production on marginal lands. *Journal of Experimental Botany*.

[2] Nobel P. S. (1991). Achievable productivities of certain CAM plants: basis for high values compared with C_3 and C_4 plants. *New Phytologist*.

[3] Yang X., Cushman J. C., Borland A. M. (2015). A roadmap for research on crassulacean acid metabolism (CAM) to enhance sustainable food and bioenergy production in a hotter, drier world. *New Phytologist*.

[4] Cedeño M. (1995). Tequila production. *Critical Reviews in Biotechnology*.

[5] Corbin K. R., Byrt C. S., Bauer S. (2015). Prospecting for energy-rich renewable raw materials: *Agave* leaf case study. *PLoS One*.

[6] Lock G. (1962). *Sisal*.

[7] Gentry S. H. (1982). *Agaves of Continental North America*.

[8] Nobel P. S., Hartsock T. L. (1986). Temperature, water, and PAR influences on predicted and measured productivity of *Agave deserti* at various elevations. *Oecologia*.

[9] Stewart J. R. (2015). *Agave* as a model CAM crop system for a warming and drying world. *Frontiers in Plant Science*.

[10] Cortés-Romero C., Martínez-Hernández A., Mellado-Mojica E., López M. G., Simpson J. (2012). Molecular and functional characterization of novel fructosyltransferases and invertases from *Agave tequilana*. *PLoS One*.

[11] Suarez-Gonzalez E. M., Lopez M. G., Délano-Frier J. P., Gómez-Leyva J. F. (2014). Expression of the 1-SST and 1-FFT genes and consequent fructan accumulation in *Agave tequilana* and *A. inaequidens* is differentially induced by diverse (a)biotic-stress related elicitors. *Journal of Plant Physiology*.

[12] Nobel P. S. (1988). *Environmental Biology of Agaves and Cacti*.

[13] Chen P. Y., Chen C. H., Kuo C. C., Lee T. H., Kuo Y. H., Lee C. K. (2011). Cytotoxic steroidal saponins from *Agave sisalana*. *Planta Medica*.

[14] Santos J. D., Vieira I. J., Braz-Filho R., Branco A. (2015). Chemicals from *Agave sisalana* biomass: isolation and identification. *International Journal of Molecular Sciences*.

[15] Good-Avila S. V., Souza V., Gaut B. S., Eguiarte L. E. (2006). Timing and rate of speciation in Agave (Agavaceae). *Proceedings of the National Academy of Sciences of the United States of America*.

[16] Robert M. L., Lim K. Y., Hanson L. (2008). Wild and agronomically important *Agave* species (Asparagaceae) show proportional increases in chromosome number, genome size, and genetic markers with increasing ploidy. *Botanical Journal of the Linnean Society*.

[17] Guo S., Zhang J., Sun H. (2013). The draft genome of watermelon (*Citrullus lanatus*) and resequencing of 20 diverse accessions. *Nature Genetics*.

[18] Lu L., Shao D., Qiu X. (2013). Natural variation and artificial selection in four genes determine grain shape in rice. *The New Phytologist*.

[19] Qi J., Liu X., Shen D. (2013). A genomic variation map provides insights into the genetic basis of cucumber domestication and diversity. *Nature Genetics*.

[20] Canales J., Bautista R., Label P. (2014). De novo assembly of maritime pine transcriptome: implications for forest breeding and biotechnology. *Plant Biotechnology Journal*.

[21] Huang X., Chen J., Bao Y. (2014). Transcript profiling reveals auxin and cytokinin signaling pathways and transcription regulation during in vitro organogenesis of ramie (*Boehmeria nivea* L. gaud). *PLoS One*.

[22] Liu T., Tang S., Zhu S., Tang Q., Zheng X. (2014). Transcriptome comparison reveals the patterns of selection in domesticated and wild ramie (*Boehmeria nivea* L. gaud). *Plant Molecular Biology*.

[23] Gross S. M., Martin J. A., Simpson J., Abraham-Juarez M., Wang Z., Visel A. (2013). *De novo* transcriptome assembly of drought tolerant CAM plants, *Agave deserti* and *Agave tequilana*. *BMC Genomics*.

[24] Wang P., Gao J., Yang F. (2014). Transcriptome of sisal leaf pretreated with Phytophthora nicotianae Breda. *Chinese J Tropical Crops*.

[25] Tamura K., Peterson D., Peterson N., Stecher G., Nei M., Kumar S. (2011). MEGA5: molecular evolutionary genetics analysis using maximum likelihood, evolutionary distance, and maximum parsimony methods. *Molecular Biology and Evolution*.

[26] Martin J., Bruno V. M., Fang Z. (2010). Rnnotator: an automated *de novo* transcriptome assembly pipeline from stranded RNA-seq reads. *BMC Genomics*.

[27] Zhang M., Leong H. W. (2010). Bidirectional best hit *r*-window gene clusters. *BMC Bioinformatics*.

[28] Doron-Faigenboim A., Stern A., Mayrose I., Bacharach E., Pupko T. (2005). Selecton: a server for detecting evolutionary forces at a single amino-acid site. *Bioinformatics*.

[29] Yang Z. (2007). PAML 4: phylogenetic analysis by maximum likelihood. *Molecular Biology and Evolution*.

[30] Li B., Dewey C. N. (2011). RSEM: accurate transcript quantification from RNA-Seq data with or without a reference genome. *BMC Bioinformatics*.

[31] Hu M., Hu W., Xia Z., Zhou X., Wang W. (2016). Validation of reference genes for relative quantitative gene expression studies in cassava (*Manihot esculenta* Crantz) by using quantitative real-time PCR. *Frontiers in Plant Science*.

[32] Librado P., Rozas J. (2009). DnaSP v5: a software for comprehensive analysis of DNA polymorphism data. *Bioinformatics*.

[33] Biasini M., Bienert S., Waterhouse A. (2014). SWISS-MODEL: modelling protein tertiary and quaternary structure using evolutionary information. *Nucleic Acids Research*.

[34] Hughes C. E., Govindarajulu R., Robertson A., Filer D. L., Harris S. A., Bailey C. D. (2007). Serendipitous backyard hybridization and the origin of crops. *Proceedings of the National Academy of Sciences of the United States of America*.

[35] Tamayo-Ordonez M. C., Rodriguez-Zapata L. C., Narvaez-Zapata J. A. (2016). Morphological features of different polyploids for adaptation and molecular characterization of CC-NBS-LRR and LEA gene families in *Agave* L.. *Journal of Plant Physiology*.

[36] Gao J., Luoping, Guo C. (2012). AFLP analysis and zebra disease resistance identification of 40 sisal genotypes in China. *Molecular Biology Reports*.

[37] Castilhos G., Lazzarotto F., Spagnolo-Fonini L., Bodanese-Zanettini M. H., Margis-Pinheiro M. (2014). Possible roles of basic helix–loop–helix transcription factors in adaptation to drought. *Plant Science*.

[38] Ciftci-Yilmaz S., Mittler R. (2008). The zinc finger network of plants. *Cellular and Molecular Life Sciences*.

[39] Dietz K. J., Vogel M. O., Viehhauser A. (2010). AP2/EREBP transcription factors are part of gene regulatory networks and integrate metabolic, hormonal and environmental signals in stress acclimation and retrograde signalling. *Protoplasma*.

[40] Dubos C., Stracke R., Grotewold E., Weisshaar B., Martin C., Lepiniec L. (2010). MYB transcription factors in *Arabidopsis*. *Trends in Plant Science*.

[41] Puranik S., Sahu P. P., Srivastava P. S., Prasad M. (2012). NAC proteins: regulation and role in stress tolerance. *Trends in Plant Science*.

[42] Singh K., Foley R. C., Onate-Sanchez L. (2002). Transcription factors in plant defense and stress responses. *Current Opinion in Plant Biology*.

[43] Kooyers N. J. (2015). The evolution of drought escape and avoidance in natural herbaceous populations. *Plant Science*.

[44] Dios E. A. D., Vargas A. D. G., Santos M. L. D., Simpson J. (2015). New insights into plant glycoside hydrolase family 32 in Agave species. *Frontiers in Plant Science*.

[45] Didi V., Jackson P., Hejatko J. (2015). Hormonal regulation of secondary cell wall formation. *Journal of Experimental Botany*.

[46] Kim W. C., Kim J. Y., Ko J. H., Kang H., Kim J., Han K. H. (2014). AtC3H14, a plant-specific tandem CCCH zinc-finger protein, binds to its target mRNAs in a sequence-specific manner and affects cell elongation in *Arabidopsis thaliana*. *The Plant Journal*.

[47] Wang D., Qin Y., Fang J. (2016). A missense mutation in the zinc finger domain of OsCESA7 deleteriously affects cellulose biosynthesis and plant growth in rice. *PLoS One*.

[48] Qin Y. M., Hu C. Y., Pang Y., Kastaniotis A. J., Hiltunen J. K., Zhu Y.-X. (2007). Saturated very-long-chain fatty acids promote cotton fiber and *Arabidopsis* cell elongation by activating ethylene biosynthesis. *The Plant Cell*.

[49] Behr M., Legay S., Žižková E. (2016). Studying secondary growth and bast fiber development: the hemp hypocotyl peeks behind the wall. *Frontiers in Plant Science*.

[50] Chantreau M., Chabbert B., Billiard S., Hawkins S., Neutelings G. (2015). Functional analyses of cellulose synthase genes in flax (*Linum usitatissimum*) by virus-induced gene silencing. *Plant Biotechnology Journal*.

[51] Chen J., Pei Z., Dai L. (2014). Transcriptome profiling using pyrosequencing shows genes associated with bast fiber development in ramie (*Boehmeria nivea* L.). *BMC Genomics*.

[52] Schubert A. M., Benedict C. R., Berlin J. D., Kohel R. J. (1973). Cotton fiber development-kinetics of cell elongation and secondary wall thickening. *Crop Science*.

[53] Cosgrove D. J. (2005). Growth of the plant cell wall. *Nature Reviews Molecular Cell Biology*.

[54] Hussey S. G., Mizrachi E., Creux N. M., Myburg A. A. (2013). Navigating the transcriptional roadmap regulating plant secondary cell wall deposition. *Frontiers in Plant Science*.

[55] Schuetz M., Smith R., Ellis B. (2012). Xylem tissue specification, patterning, and differentiation mechanisms. *Journal of Experimental Botany*.

[56] Nelson C. J., Spollen W. G. (1987). Fructan. *Physiologia Plantarum*.

[57] Valluru R., Ende W. V. D. (2008). Plant fructans in stress environments: emerging concepts and future prospects. *Journal of Experimental Botany*.

[58] Abraham P. E., Yin H., Borland A. M. (2016). Transcript, protein and metabolite temporal dynamics in the CAM plant *Agave*. *Nature Plants*.

